# Effect of Whole-Body Cryotherapy on Oxidant–Antioxidant Imbalance in Women with Multiple Sclerosis

**DOI:** 10.3390/jcm12185958

**Published:** 2023-09-13

**Authors:** Bartłomiej Ptaszek, Szymon Podsiadło, Justyna Adamiak, Jakub Marchewka, Łukasz Tota, Aneta Teległów

**Affiliations:** 1 Institute of Applied Sciences, University of Physical Education in Krakow, 31-571 Krakow, Poland; justyna.adamiak@awf.krakow.pl; 2 Institute of Clinical Rehabilitation, University of Physical Education in Krakow, 31-571 Krakow, Poland; szymon.podsiadlo@awf.krakow.pl (S.P.); jakub.marchewka@awf.krakow.pl (J.M.); 3 Institute of Biomedical Sciences, University of Physical Education in Krakow, 31-571 Krakow, Poland; lukasz.tota@awf.krakow.pl; 4 Institute of Basic Sciences, University of Physical Education in Krakow, 31-571 Krakow, Poland; aneta.teleglow@awf.krakow.pl

**Keywords:** cryotherapy, oxidative stress, free radicals, multiple sclerosis, neurons, antioxidant capacity

## Abstract

The aim of the study was to investigate whether 20 whole-body cryotherapy treatments have an effect on oxidative–antioxidant imbalances in women with multiple sclerosis. Fifty women aged 30–55 were examined: study group—15 women with multiple sclerosis, subjected to whole-body cryotherapy; first control group—20 women with multiple sclerosis who did not receive cryotherapy intervention; second control group—15 healthy women who participated in cryotherapy treatments. Blood from the examined women was collected twice (before and after the series of 20 cryotherapy sessions). An insignificant increase in the total antioxidant capacity (study group: *p* = 0.706; second control group: *p* = 0.602) was observed after the whole-body cryotherapy intervention. After the series of cryotherapy sessions, the total oxidative status/total oxidative capacity value was insignificantly decreased among the multiple sclerosis patients (decrease by 14.03%, *p* = 0.495). In women with multiple sclerosis, no significant cryotherapy impact was demonstrated on changes in the oxidant–antioxidant imbalance or concentrations of nitric oxide, uric acid, or matrix metalloproteinase-9.

## 1. Introduction

Multiple sclerosis (MS) is a demyelinating disease of the central nervous system (CNS). The etiology of this multifactorial disease has not been clearly defined [1]. The more frequent occurrence of MS among women is evident in all studies conducted worldwide. In Europe, women are affected twice as often as men [2]. This may be related to the higher incidence of viral diseases in women or simply to the larger number of women in the population [3].

Rehabilitation of individuals with MS should be implemented at every stage of the disease and performed continuously, not only in the hospital setting [4]. Complex rehabilitation should address physical, psychological, and social problems [5]. Physiotherapy treatment includes physical rehabilitation, aimed at developing muscle strength, reducing spasticity, as well as improving motor coordination, balance, and postural reflexes. Of considerable importance also is psychological rehabilitation, intended to provide psychological support to the patient in the new health situation, and social and vocational rehabilitation, focused on helping the individual to adapt to the new situation at work [6].

The analysis of cryogenic temperatures’ impact on human thermoregulation processes is essential from the point of view of both the safety of the applied procedures and scientific research. Maintaining thermal balance is one of the fundamental physiological mechanisms responsible for the proper function of the human body, even in widely varying environments [7]. Whole-body cryotherapy (WBC) is used to induce physiological, systemic body defense responses that are beneficial and effective in maintaining or restoring homeostasis [8,9]. WBC is a method to raise the threshold of pain or to eliminate pain [10]. The body’s reaction to cold involves changes in many systems, mainly cardiovascular, neuromuscular, as well as immune and endocrine [11,12,13].

Previous research shows that cooling the body improves locomotion, reduces bladder dysfunction and muscle spasticity, reduces fatigue and pain, and improves muscle strength, coordination, vision, and cognitive abilities of people with MS [14,15,16,17]. Reynolds et al. (2011) confirmed the positive effect of local cooling of the head and neck area on the functional status of people with MS [18]. Rice et al. (2009) showed that cooling the quadriceps muscle improves conduction in the femoral nerve and bioelectrical activity of the cooled muscle [19]. After a series of treatments in a cryogenic chamber, a clear functional improvement is also observed resulting from the reduction of spasticity and the improvement of body stability in a standing position [20].

The aim of the study was to investigate whether 20 whole-body cryotherapy treatments have an effect on oxidative–antioxidant imbalances in women with multiple sclerosis.

## 2. Material and Methods

### 2.1. Participant Characteristics

The presented study followed the tenets of the Declaration of Helsinki. Approval was obtained from the Bioethics Committee of the Regional Medical Chamber in Krakow (87/KBL/OIL/2018, 8 May 2018). The trials are also registered with the Australian New Zealand Clinical Trials Registry (ACTRN12620001142921, 2 November 2020). Overall, 80 women applied to the study, and 50 were finally qualified to participate in the research program. Participants underwent medical and physiotherapeutic qualifications (general assessment of the health status of the subjects, functional status, stage, course and type of disease, determination of the applied treatment). The inclusion and exclusion criteria are provided in Table 1. The research is a continuation of our previous research on the impact of WBC in MS patients and concerns the same group of patients [21,22].

50 women were qualified for the study:

- 15 women aged 34–55 years with diagnosed MS + 20 WBC treatments—the study group (CRYO-MS);
- 20 women aged 32–48 years with diagnosed MS + no WBC intervention. Nonprobability sampling was applied. The patients had no contraindications to WBC but could not use WBC due to constraints (work, family, …)—the first control group (CONTROL-MS);
- 15 healthy women aged 30–49 (without chronic diseases, including neurological ones) + 20 WBC treatments—the second control group (CONTROL-CRYO).

The exact, general characteristics of the investigated women were presented in our previous publication [21]. The Tanita BC 418 MA (body weight, body composition) and measuring tape (body height) were used for the measurements.

### 2.2. Analysis of Biochemical Blood Indices

For the analysis of biochemical blood indices, venous blood was collected twice: on the day of WBC commencement and after the series of 20 cryotherapy sessions. There was one-time (at baseline) examination of women without WBC intervention. Fasting blood samples were collected (by a qualified laboratory diagnostician in accordance with applicable standards) in the morning from the basilic, cephalic, or median cubital vein into test tubes with a clotting activator—for serum testing (6 mL). The total antioxidant status/total antioxidant capacity (TAS/TAC) and the total oxidative status/total oxidative capacity (TOS/TOC) were determined in the plasma. The indices were investigated with photometric tests (TAS/TAC: ImAnOX kit, KC5200; TOS/TOC: PerOx kit, KC5100; Immundiagnostik AG, Bensheim, Germany) (sample volume: 10 µL, detection limit: 130 µmol/L, reading: 450 nm). Nitric oxide (NO) and uric acid (UA) concentrations were also established in the study participants. Serum NO concentration was determined with the spectrophotometric method and a Nitrate/Nitrite Colorimetric Assay Kit 0560404 (Cayman Chemical Company, Ann Arbor, MI, USA) (reading: 450 nm). UA concentration was investigated with a Cobas 6000 analyzer (Roche, Basel, Switzerland), applying the enzymatic method with uricase and peroxidase (reading: 530–570 nm). Matrix metalloproteinase-9 (MMP-9) assessment was performed with the immunoenzymatic method, with a Human MMP-9 ELISA kit (DRG Instruments GmbH, EIA 4861, Marburg, Germany) (reading: 450 nm).

### 2.3. Description of the Intervention

WBC procedures were performed daily (Monday–Friday; 15:00–17:00) in a Wrocław-type cryochamber; refrigerant cooling: liquid nitrogen; concentration of oxygen in the air: 21–22%; chamber temperature: −120 °C; atrium temperature: −60 °C; WBC treatments duration: 3 min. In total, they all received 20 treatments. Women entering the cryochamber were dressed in accordance with the rules applicable during WBC procedures. The conditions inside the chamber were the same during all treatments in the series. After each treatment, there was a warm-up for each patient on a cycle ergometer (Kettler Corsa, KETTLER Holding GmbH, Frankfurt am Main, Germany) without resistance for 15 min.

### 2.4. Statistical Analysis

The data presented in the paper were described using the mean and standard deviation or using the median and I and III quartiles. The presentation of the data depended on the distribution of the variables (normal or non-normal distribution). The Shapiro–Wilk test was used to assess the distribution of variables. The observed differences between the experimental group and the control groups were verified using one-way analysis of variance (ANOVA). If the assumptions for the application of the above-mentioned test were not met, the Kurskal–Wallis test was used. Depending on the size of the groups, Tukey’s test (for unequal size) or Dunn’s test were used to assess the post-hoc effects. Depending on the distribution of variables, the Student’s *t*-test or Wilcoxon’s test was used for dependent variables, and the Student’s *t*-test or Mann–Whitney U test for unrelated variables. The level of statistical significance was assumed at *p* = 0.05. The hypotheses were verified bilaterally. In order to determine the sample size, the minimum sample size formula was used. Criteria were set at the level of 95% confidence interval, 0.5 fraction size, and 5% maximum error. The Statistica 13 package (Tibco Software Inc., Palo Alto, CA, USA) was used.

## 3. Results

In TAC analysis, an increase in the TAS/TAC concentration was observed after the WBC application in the groups who received the intervention (CRYO-MS and CONTROL-CRYO). However, the change was statistically insignificant (CRYO-MS: *p* = 0.706; CONTROL-CRYO: *p* = 0.602). There were no statistically significant between-group differences in baseline levels. An insignificant decrease in the TOS/TOC concentration was revealed among MS patients (14.03%, *p* = 0.495). There was also an insignificant increase in the TOS/TOC concentration after WBC in the healthy women (45.31%, *p* = 0.233). The insignificance of the changes was caused by too-wide dispersions of individual results among the subjects (Table 2, Table 3 and Table 4).

## 4. Discussion

The present study attempted to evaluate the effects of a series of 20 WBC sessions on oxidant–antioxidant imbalance and selected disease markers in women with MS. Given the problems encountered in the physiotherapeutic evaluation of MS patients, there is a permanent need for continued research into the effectiveness of different types of therapies and physiotherapeutic methods, their cost-effectiveness, and the appropriate timing of implementation [23]. There are reports in the literature that early physiotherapy commencement enhances the effects of pharmacological management, which may support the need for concomitant pharmacological and physiotherapeutic interventions in MS [24].

Oxidative stress is indicated as one of the factors involved in MS pathogenesis, especially because of the involvement of reactive oxygen species in demyelinating processes [25]. In previous research, MS patients were characterized by higher values of oxidative stress indicators [26,27] and the presence of DNA damage dependent on reactive oxygen species and associated with neurodegeneration [28,29]. The present study did not reveal significant changes in TAS/TAC concentration between the study group and the control groups. The baseline TOS/TOC concentration was lower among healthy women, although the difference was statistically insignificant (*p* = 0.293).

Recent clinical trials have demonstrated the antioxidant impact of WBC in MS patients. One must distinguish, however, between a single exposure to cryogenic temperatures, which is most likely an oxidant factor, and the use of at least 10 WBC sessions, which may constitute an antioxidant factor [30,31].

In clinical trials conducted among patients with secondary progressive MS (without relapse episodes) and with progressively worsening disability, a statistically significant increase in TAS/TAC levels was observed after 10 WBC sessions. Miller et al. [32] investigated TAS/TAC concentration, as well as superoxide dismutase (SOD) and catalase (CAT) activity in MS patients participating in kinesitherapy and a series of 10 WBC sessions, MS patients participating only in kinesitherapy sessions, and a control group of healthy subjects. The indices in MS patients showed increased oxidative stress, higher than in healthy subjects, which was reduced in both the exercise-only group and in the group participating in kinesiotherapy and receiving WBC treatment. It seems that the observed low plasma TAS/TAC levels in MS patients may also be dependent on low concentrations of endogenous antioxidants, mainly UA [30].

Another study by Miller et al. [33] determined plasma TAS/TAC levels and SOD and CAT activity in erythrocytes of MS patients before and after 10 WBC sessions (–20 °C, 3 min per day). In a subgroup of participants, the 10 cryotherapy exposure sessions were combined with the application of 10 mg melatonin. The study demonstrated that WBC markedly altered the TAS/TAC level in MS patients: in both groups (with and without melatonin) it was significantly higher after WBC than before the intervention. CAT activity was significantly (2-fold) higher in erythrocytes of MS patients than those of healthy individuals. SOD and CAT activities in erythrocytes of MS patients did not change after the WBC treatment. Melatonin supplementation increased SOD and CAT activity compared with the no-supplementation group but had no effect on TAS/TAC levels in MS patients. The authors suggest that WBC may inhibit oxidative stress in MS patients.

Miller et al. [34] implied that cryostimulation could reduce oxidative stress not only by a significant increase in TAS/TAC levels, but also by an induced elevation of SOD and glutathione peroxidase activity in erythrocytes of healthy individuals. WBC appears to improve the body’s antioxidant capacity. Further studies are needed to elucidate antioxidant mechanisms in humans and to determine the short- and long-term effects of cryogenic chamber application. The present analysis of the WBC effect on the total antioxidant potential of the body revealed an increase in TAS/TAC concentrations after WBC. This change, however, was not statistically significant. Similarly, the analysis of the total oxidant potential of the body showed reduced TOS/TOC levels after WBC in female MS patients (14.03%, *p* = 0.495), although the change also turned out statistically insignificant because of too wide dispersions of individual results among the subjects. These data do not clearly support previous findings in this area, perhaps owing to the use of 20 rather than 10 WBC sessions.

NO is a free radical discovered as a signaling molecule: it regulates, among others, blood vessel dilation and neuronal function. Circumstantial evidence suggests that NO plays a role in several features of the disease (damage and demyelination of oligodendrocytes, axonal degeneration) and contributes to loss of function by impairing axonal conduction [35]. The potential involvement of NO in MS etiology was proposed after the discovery of its critical role in inflammation [36]. Numerous experimental studies have shown elevated concentrations of NO not only in the serum but also in the urine and cerebrospinal fluid of MS patients. Serum NO concentrations have also been demonstrated to depend on the type of MS and the relapse rate [35,37,38,39].

In MS, neuronal cells in inflammatory foci may become hypoxic; this happens because inflammatory cells, as a result of inflammation, produce large amounts of NO [35], which inhibits electron transport in mitochondria and, thus, blocks oxidative phosphorylation [40,41]. Although there is some controversy on this issue, most studies regarding the involvement of NO in MS report higher NO activity in MS patients compared with controls. Increased serum NO concentrations in MS may also result from a physiological response to general oxidative stress [42]. Beneficial effects of suit cooling in MS patients were observed in the form of decreased NO production. These findings suggest that a decrease of NO concentrations improves the conduction block in demyelinated axonal segments after a cooling procedure in MS [43]. In the present study, the analysis of NO concentrations did not indicate between-group differences at baseline (MS patients and healthy women) or a statistically significant impact of WBC. Only a trend towards lower NO concentrations after WBC in MS patients was observed.

UA is the main endogenous non-enzymatic antioxidant in the human body [44]. Hooper et al. [45] were the first to report lower serum UA concentrations in MS patients compared with a control group that included mainly patients with spinal cord injury or Parkinson’s disease. They found that MS and gout were mutually exclusive diseases, suggesting that hyperuricemia might protect against MS. It has been suggested that reduced UA concentration in MS represents a primary constitutive loss of protection against oxidative stress [46] and that high concentrations of UA, as a powerful antioxidant, may reduce the risk of MS development or decrease its progression [47]. Mostert et al. [48] concluded that MS patients were not deficient in serum UA, that their serum UA concentrations decreased only during relapses, and that increasing UA concentrations could provide a therapeutic benefit; no correlation was described between UA concentrations and the course of the disease or the use of immunomodulating therapy.

Massa et al. [49] conducted a prospective study to assess whether serum UA concentrations could be used to predict MS risk; however, they found that UA was not a strong predictor of MS risk and that the lower UA levels among MS cases were a consequence rather than a cause of the disease. UA is a potent antioxidant observed in extracellular fluid and is thought to account for more than half of plasma antioxidant capacity. It has been suggested that UA may exert neuroprotective effects as a scavenger of reactive nitrogen and oxygen radicals. A potent antioxidant impact of UA on neurons has been demonstrated in in vivo and in vitro studies [50]. Miller et al. [51] advocated that UA could serve as an easily detectable marker of disease activity and response to treatment. Some studies have evaluated the correlation between UA concentrations and disease activity, disability, disease course, or disease duration. Karg et al. [52] showed no relationship between UA concentration and the disease clinical activity or immunomodulating treatment, and Peng et al. [53,54] stated that UA concentration did not correlate with the disease MRI activity, disease subtype, or disability.

Subsequent studies, however, revealed numerous correlations. Sotgiu et al. [46] indicated that serum UA concentrations were lower in MS patients with a more disabling disease (EDSS score > 3.5). Guerrero et al. [55] implied significantly lower UA concentrations during relapses, correlating with the degree of disability of MS patients as assessed with the EDSS scale. You et al. [56] and Moccia et al. [57] reported that patients with neuromyelitis optica and MS with an EDSS score > 5 had lower serum UA concentrations than those with an EDSS score < 5. This suggests that serum UA concentrations are closely related with MS disability and neuromyelitis optica. Similar results were obtained among patients in remission and those in relapse. Patients in relapse had lower serum UA concentrations than those in remission. Thus, serum UA concentration may be a potential biomarker of MS-related disability and neuromyelitis optica. A neuroprotective potential of UA has been speculated [58].

The present study also showed lower baseline UA concentrations in MS women (in the study group and in the control group). Despite mean differences of 37.15 µmol/L, these changes did not reach statistical significance (*p* = 0.110).

The purpose of another study by Miller et al. [59] was to determine the long-term effects of 10 sessions of WBC on plasma UA concentrations in selected patients with secondary progressive MS and to verify the results with the functional status of the patients as assessed by EDSS. The research revealed that WBC had long-lasting effects on the concentration of the major antioxidant in human blood, UA, both immediately after completing the intervention and 1 and 3 months later on. It appears that WBC, by improving the functional status and increasing blood UA concentrations, may be applied as an adjunctive treatment in MS. Although the authors did not find a statistically significant correlation between plasma UA concentrations and the degree of disability in MS patients, they observed a trend toward a negative correlation.

Lowering body temperature can increase the conduction of nerve signals and alleviate many symptoms, especially fatigue [60]. Miller et al. [59] also observed long-term effects of WBC on UA concentrations in MS patients. The disability status was assessed by using a perceptual scale, and the results showed a reduction in disability during WBC treatment. In the present study, no statistically significant changes in UA concentrations were noticed after 20 WBC sessions in either women with MS or healthy women.

Studies of MS patients’ brain tissue, serum, and cerebrospinal fluid consistently showed increased MMP levels. MMP-2 and MMP-9 have been studied in greater detail in MS [61]. Elevated MMP levels were detected in brain tissue collected from individuals who died with MS [62]. Serum MMP-9 has been typically observed in patients with active disease, manifested by continuous clinical relapse [63].

Levels of the active form of MMP-9 were elevated in the cerebrospinal fluid of MS patients, particularly during active disease, compared with samples of control subjects with inflammatory neurological diseases and healthy controls [64]. Liuzzi et al. [65] indicated that the cerebrospinal fluid MMP-9 level was increased among individuals with active relapsing-remitting MS compared with stable patients. Experimental findings showed intrathecal synthesis of MMP-9 in MS and its increased levels in the cerebrospinal fluid and serum of relapsing-remitting MS patients compared with primary progressive and healthy control subjects [65,66,67].

Moreover, some authors have reported that higher plasma MMP-9 levels are associated with disease severity. There is evidence linking both MMP and Th17/Th1-related cytokines with MS pathogenesis [68]. Both protein families have been referred to as markers of disease activity [69].

In the present study, we obtained contrary results, showing higher baseline levels in healthy women compared with the control group with MS (*p* = 0.017) and with the study group with MS (statistically insignificant difference, *p* = 0.249). We also observed a statistically insignificant decrease in MMP-9 in healthy women after WBC application (from 437.98 ± 108.43 ng/mL to 385.96 ± 88.63 ng/mL) but the intervention did not significantly impact on the level of MMP-9 in females with MS.

## 5. Conclusions

In women with MS, no significant cryotherapy impact was demonstrated on changes in the oxidant–antioxidant imbalance or concentrations of NO, UA, or MMP-9. However, the research should continue in larger groups and in different forms of MS.

## 6. Study Limitation

The current experiment has minor flaws related to the small number of participants, the lack of dietary control, or the lack of careful monitoring of the daily schedule and activity of the participants.

## Figures and Tables

**Table 1:** Inclusion and exclusion criteria for the study group and the first control group.

Inclusion Criteria	Exclusion Criteria
Female sex Age: 30–55 years Diagnosed MS—McDonald review criteria (the MS groups) Expanded Disability Status Scale (EDSS) score of 0–6.5 Written consent to participate in the study	Contraindications to WBC Consuming more than 4 cups of coffee or more than 2 alcoholic beverages per day Changing the diet immediately before or during the study Participation in other forms of physical activity directly before or during the study

**Table 2:** Oxidant–antioxidant balance and selected disease markers before the intervention sessions.

Parameter	CRYO-MS (*n* = 15)	CONTROL-MS (*n* = 20)	CONTROL-CRYO (*n* = 15)	*p* (ANOVA)
TAS/TAC [µmol/L]	257.53 ± 30.57	265.85 ± 38.20	255.07 ± 30.81	0.613
TOS/TOC [µmol/L]	1062.0 (711.0–1364.0)	1095.0 (884.5–1475.5)	512.0 (372.0–1400.0)	0.293
NO [µM]	15.0 (11.0–17.0)	14.5 (10.5–18.5)	15.0 (8.0–24.0)	0.418
UA [µmol/L]	240.12 ± 62.45	236.42 ± 55.61	275.42 ± 51.60	0.110
MMP-9 [ng/mL]	385.05 ± 67.23	344.50 ± 89.12	437.98 ± 108.43	0.014 *
	*p* (CRYO-MS/CONTROL-MS) 0.437	*p* (CONTROL-MS/CONTROL-CRYO) 0.249	*p* (CRYO-MS/CONTROL-CRYO) 0.017	

CRYO-MS: the group of women with diagnosed MS who underwent a series of WBC sessions; CONTROL-MS: the first control group, women with diagnosed MS who received no cryotherapy intervention; CONTROL-CRYO: the second control group, healthy women who participated in WBC sessions; ANOVA: analysis of variance; TAS/TAC: total antioxidant status/total antioxidant capacity; TOS/TOC: total oxidative status/total oxidative capacity; NO: nitric oxide; UA: uric acid; MMP-9: matrix metalloproteinase-9; *: statistically significant (*p* < 0.05).

**Table 3:** Oxidant–antioxidant balance and selected disease markers after the intervention sessions.

Parameter	CRYO-MS (*n* = 15)	CONTROL-MS (*n* = 20)	CONTROL-CRYO (*n* = 15)	*p* (ANOVA)
TAS/TAC [µmol/L]	261.00 ± 44.87	265.85 ± 38.20	259.80 ± 39.69	0.895
TOS/TOC [µmol/L]	913.0 (535.0–1180.0)	1095.0 (884.5–1475.5)	744.0 (441.0–1313.0)	0.465
NO [µM]	12.0 (10.0–22.0)	14.5 (10.5–18.5)	17.0 (10.0–29.0)	0.488
UA [µmol/L]	229.47 ± 52.70	236.42 ± 55.61	275.46 ± 65.08	0.069
MMP-9 [ng/mL]	378.83 ± 61.28	344.50 ± 89.12	385.96 ± 88.63	0.277

CRYO-MS: the group of women with diagnosed MS who underwent a series of WBC sessions; CONTROL-MS: the first control group, women with diagnosed MS who received no cryotherapy intervention; CONTROL-CRYO: the second control group, healthy women who participated in WBC sessions; ANOVA: analysis of variance; TAS/TAC: total antioxidant status/total antioxidant capacity; TOS/TOC: total oxidative status/total oxidative capacity; NO: nitric oxide; UA: uric acid; MMP-9: matrix metalloproteinase-9.

**Table 4:** Changes in oxidant–antioxidant balance indices and selected disease markers after the intervention sessions.

Parameter	Before	After	*p* (*t*-Student/Wilcoxon)
CRYO-MS (*n* = 15)			
TAS/TAC [µmol/L]	257.53 ± 30.57	261.00 ± 44.87	0.706
TOS/TOC [µmol/L]	1062.0 (711.0–1364.0)	913.0 (535.0–1180.0)	0.495
NO [µM]	15.0 (11.0–17.0)	12.0 (10.0–22.0)	0.594
UA [µmol/L]	240.12 ± 62.45	229.47 ± 52.70	0.191
MMP-9 [ng/mL]	385.05 ± 67.23	378.83 ± 61.28	0.745
CONTROL-CRYO (*n* = 15)			
TAS/TAC [µmol/L]	255.07 ± 30.81	259.80 ± 39.69	0.602
TOS/TOC [µmol/L]	512.0 (372.0–1400.0)	744.0 (441.0–1313.0)	0.233
NO [µM]	15.0 (8.0–24.0)	17.0.(10.0–29.0)	0.379
UA [µmol/L]	275.42 ± 51.60	275.46 ± 65.08	0.997
MMP-9 [ng/mL]	437.98 ± 108.43	385.96 ± 88.63	0.078

CRYO-MS: the group of women with diagnosed MS who underwent a series of WBC sessions; CONTROL-CRYO: the second control group, healthy women who participated in WBC sessions; TAS/TAC: total antioxidant status/total antioxidant capacity; TOS/TOC: total oxidative status/total oxidative capacity; NO: nitric oxide; UA: uric acid; MMP-9: matrix metalloproteinase-9.

## Data Availability

All data generated or analyzed during this study are included in this published article.
